# Network Pharmacology-Based Strategy and Molecular Docking to Explore the Potential Mechanism of Jintiange Capsule for Treating Osteoporosis

**DOI:** 10.1155/2021/5338182

**Published:** 2021-12-03

**Authors:** Zhao Yang, Zhen-Zhen Yuan, Xin-Long Ma

**Affiliations:** Department of Orthopedics, Tianjin Hospital, Tianjin 300211, China

## Abstract

**Background:**

With the advent of ageing population, osteoporosis (OP) has already become a global challenge. Jintiange capsule is extensively applied to treat OP in China. Although recent studies demonstrate that it generates significant effects on strengthening bone, the exact mechanism of the jintiange capsule for treating OP remains unknown.

**Purpose:**

To understand the main ingredients of the jintiange capsule, predict the possible targets and the relevant signal transduction pathways, and explore the mechanism of the jintiange capsule for the treatment of OP.

**Methods:**

Main ingredients of the jintiange capsule, drug targets, and potential disease targets for OP were obtained from public databases. Molecular biological processes and signaling pathways were determined via bioinformatic analysis, containing protein-protein interaction (PPI), Gene Ontology (GO), and Kyoto Encyclopedia of Genes and Genomes (KEGG). Subsequently, the disease-drug-ingredient-targets-pathways networks were constructed using Cytoscape. According to CytoNCA, core targets were acquired. Finally, the present study conducted molecular docking for better testing the abovementioned results.

**Results:**

In the current work, we found that 4 main ingredients of the jintiange capsule, 33 drug targets, 4745 potential disease targets for OP, and 12 overlapping targets were identified. PPI network containing 12 nodes and 25 edges proved that there existed a complex relationship. As revealed by GO functional annotation, the intersected targets were mostly associated with BP, CC, and MF. The targets were enriched to 368 items in BP, 27 items in CC, and 42 items in MF. They mainly included calcium ion homeostasis, calcium channel complex, and calcium channel regulator activity. According to KEGG pathway analysis, the intersected targets were mostly associated with Rap 1, cGMP-PKG, Ras, cAMP, calcium pathways, and so on. Based on the analysis with CytoNCA, we acquired 4 core targets, respectively—CALR, SPARC, CALM1, and CALM2. Besides, 2 core targets, CALR and CALM1, were selected for molecular docking experiments. Molecular docking revealed that the main ingredient, calcium phosphate, had good binding with the CALR protein and CALM1 protein.

**Conclusion:**

To conclude, the main ingredient of the jintiange capsule, particularly calcium phosphate, may interact with 2 targets, CALR and CALM1, and regulate multiple signaling pathways to treat OP. Additionally, this also benefits us in further understanding the mechanism of the jintiange capsule for treating OP.

## 1. Introduction

Osteoporosis (OP) refers to the systemic progressive skeletal disorder, which is featured by bone tissue microarchitectural deterioration and decreased bone density, causing fracture tendency and bone fragility [1]. The USA has witnessed over 10 million OP cases. Meanwhile, there are 200 million male and female OP cases globally. With the advent of an aging population, OP becomes the worldwide concern influencing the life quality of OP individuals [2]. Therefore, timely diagnosis and treatment of this disease are of great necessity [3].

As the valuable treasure in China, Traditional Chinese Medicine (TCM) exhibits particular effects on preventing and treating diseases [4]. Jintiange capsule, a TCM formula, is extensively applied for the treatment of OP. It contains artificial tiger bone powders prepared from diverse animal bones, whose components and therapeutic efficacy are close to those of tiger bone. However, the application of tiger bone is forbidden since tiger is among the list of protected animals in China [5]. Although recent studies also reveal that it exerts significant effects on strengthening bone, the exact mechanism of the jintiange capsule for treating OP remains unknown.

Network pharmacology (NP) is a new discipline based on the theory of system biology, which explores the network of the biological system and selects specific signal nodes to design multitarget drug molecules. First proposed by Hopkins in 2007, NP is a promising approach that can combine information science with systems medicine [6]. NP provides an efficient approach for investigating the synergy of TCM components and their related mechanisms [7]. Apart from that, molecular docking (MD) is a method of drug design through the characteristics of receptors as well as the interaction between receptors and drug molecules. It can be applied in computer-assisted drug discovery, particularly in new drug discovery to treat disorders [8]. The abovementioned two methods provide the effective way to investigate the active sites in drugs and exert important functions in TCM [9]. The present work adopted NP and MD for comprehending the major components in the jintiange capsule, predicting the possible targets and pathways as well as exploring those mechanisms of the jintiange capsule for treating OP.

## 2. Methods

### 2.1. Screening for Main Ingredients

The Traditional Chinese Medicine Integrated Database (TCMID, http://www.megabionet.org/tcmid/) was employed to obtain the main ingredients of tiger bone. TCMID is a TCM database with 842 disorders and novel connections, 18203 herbal components, 85 targets, 1356 drugs, and 15 prescriptions [10]. The present study utilized the keyword tiger bone to search the TCMID database. In addition, electronic databases PubMed, Google Scholar, and CNKI were adopted to retrieve relevant studies.

### 2.2. Screening for Drug Targets

DrugBank database (https://go.drugbank.com/) was adopted to retrieve drug targets for main ingredients. Those selected ingredients were uploaded into DrugBank database, and targets were collected.

### 2.3. Potential Disease Targets for OP

OP-associated targets were searched in three human gene databases, including Online Mendelian Inheritance in Man (OMIM, https://www.omim.org/), GeneCards (https://www.genecards.org/), and DisGeNET (https://www.disgenet.org/). Then, we combined all targets obtained in the three databases.

### 2.4. Acquisition of Overlapping Targets

The targets of tiger bone's main ingredient and potential disease targets for OP obtained from the above 3 databases were applied to obtain the overlapping targets.

### 2.5. Protein-Protein Interaction (PPI) Network Construction

STRING database (https://string-db.org/), a database that predicted known protein interactions, was utilized for constructing and visualizing the PPI network of the overlapping targets. At the same time, the custom value of STRING was set to 0.2.

### 2.6. Gene Ontology (GO) and Kyoto Encyclopedia of Genes and Genomes (KEGG) Pathway Enrichment Analysis

GO and KEGG pathway enrichment analysis were conducted for predicting targets of the jintiange capsule for treating OP by a bioconductor, a data package in R software (version 4.1.0). *q* value ≤0.05 was set. Besides, the top 10 GO enrichments and KEGG pathways with higher counts were investigated.

### 2.7. Construction of Disease-Drug-Ingredient-Targets-Pathways (D-D-I-T-P) Networks

D-D-I-T-P networks were established to comprehend the mechanism of the jintiange capsule for treating OP. The networks were constructed and visualized by Cytoscape 3.8.2 software. CytoNCA, a Cytoscape plugin, was used for performing topological analysis of the PPI network. Thus, the core targets can be obtained.

### 2.8. Molecular Docking

This study obtained 2D structures for major ingredients in PubChem database (https://pubchem.ncbi.nlm.nih.gov/). Thereafter, we converted the 2D structures to the MOL2 format by adopting Chem3D software and employed them as the ligands. Additionally, we obtained 3D structures for receptor proteins in RCSB PDB database (https://www.rcsb.org/). Subsequently, the SYBYL-X software was used to achieve MD between the receptors and ligands.

## 3. Results

### 3.1. Main Ingredients of Tiger Bone

From the TCMID database and literature, a total of 4 ingredients of tiger bone were obtained including calcium phosphate, magnesium phosphate, osseocolla, and fat, respectively.

### 3.2. Drug Targets of Main Ingredients

Totally, 33 drug targets were retrieved from DrugBank database. The targets of main ingredients are illustrated in Table 1.

### 3.3. Potential Disease Targets for OP

Based on GeneCards database, a total of 4449 potential disease targets which are known for OP were obtained. Altogether, 1098 potential disease targets were collected form DisGeNET database. Afterwards, 12 potential targets were acquired from OMIM database. After merging, we acquired 4745 potential disease targets.

### 3.4. The Acquisition of Overlapping Genes

**Figure 1:**
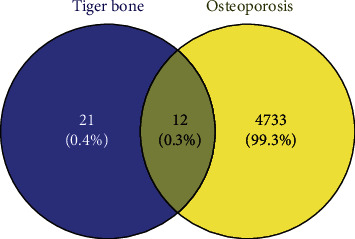
The 12 overlapping targets between the drug and the disease.

Drug targets were intersected with the disease targets to obtain 12 overlapping targets, suggesting that such targets might exert critical effects on treating OP. As shown in Figure 1, these overlapping targets include CASR, CALR, SPARC, FBN2, RGN, S100A6, TPT1, NUCB2, CALM1, FBN3, CALM2, and NRXN1.

### 3.5. PPI Network Construction

**Figure 2:**
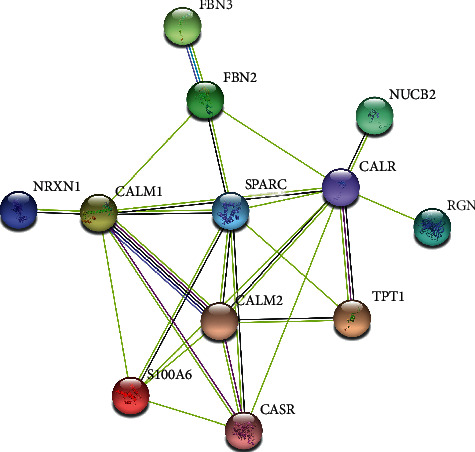
A PPI network containing 12 nodes and 25 edges.

We imported 12 overlapping targets between the drug and the disease to STRING database in order to construct the PPI network (Figure 2), containing 12 nodes and 25 edges. Based on the PPI network analysis, such targets had complicated relations.

### 3.6. GO and KEGG Pathway Enrichment Analysis

**Figure 3:**
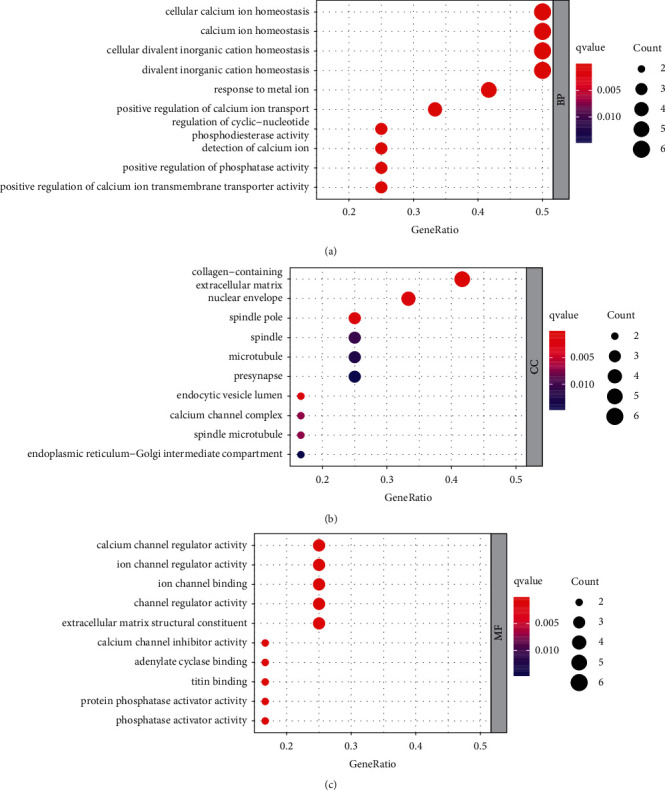
GO functional annotation of 12 intersected targets. (a) The 10 most significant BP terms. (b) The 10 most significant CC terms. (c) The 10 most significant MF terms.

To further understand the abovementioned overlapping targets, we utilized the R software Bioconductor package for GO functional annotation and KEGG pathway analysis. GO terms can be classified as the following 3 diverse categories: biological process (BP), cellular component (CC), and molecular function (MF). We selected the top 10 according to the *q* value from small to large, which can be observed in Figure 3. With a total of 368 items in BP, the targets were related to calcium ion homeostasis, divalent inorganic cation homeostasis, response to metalion, and others. The targets were enriched to 27 items in CC, which mainly included collagen-containing extracellular matrix, nuclear envelope, spindle, and calcium channel complex. Concerning 42 items in MF, it mainly contained calcium channel regulator activity, ion channel binding, cytokine activity, phosphatase activator activity, etc.

**Figure 4:**
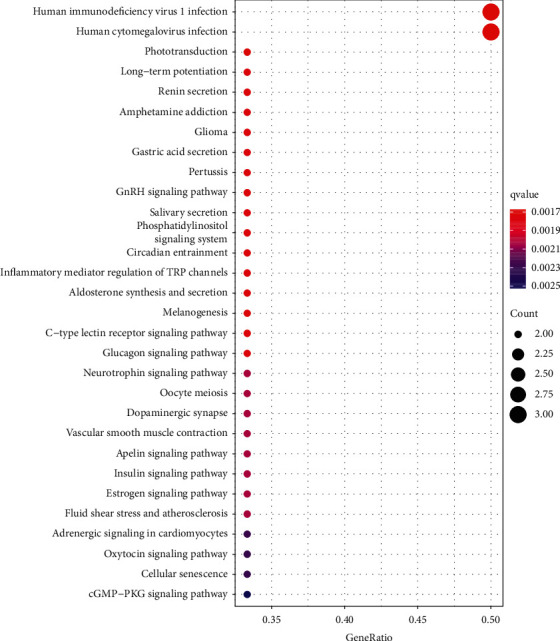
KEGG pathways for the 12 intersected targets.

A total of 45 pathways (Figure 4) were screened out via the KEGG enrichment pathway analysis including Rap1, cGMP-PKG, Ras, cAMP, and calcium pathways. There were several proteins in one pathway. At the same time, one single protein also participated in several pathways. Through the above pathways, such a regulating mechanism revealed the feasibility of using certain major tiger bone ingredients in the treatment of certain disorders, such as OP.

### 3.7. Construction of D-D-I-T-P Networks

**Figure 5:**
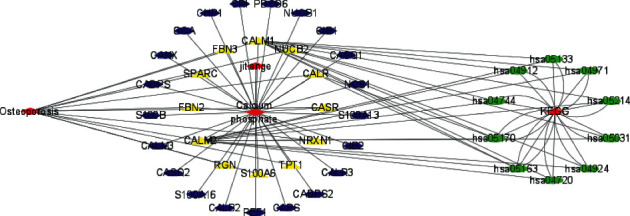
D-D-I-T-P networks.

**Figure 6:**
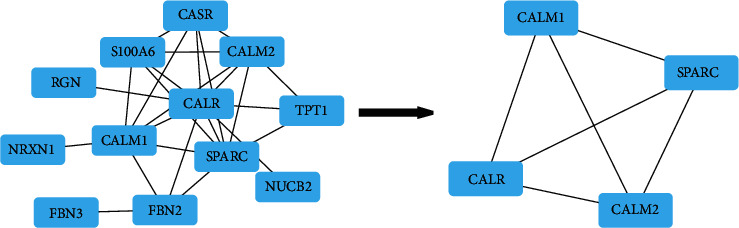
According to the analysis with CytoNCA, the core targets were obtained.

A total of 4 main ingredients were gained from tiger bone. Among 4 ingredients, 3 ingredients could not successfully predict targets. As a result, only 1 ingredient was retained. Furthermore, we chose 1 ingredient, 33 drug targets, and 10 pathways involving most targets for the construction of the D-D-I-T-P networks (Figure 5) by adopting Cytoscape software. According to the analysis with CytoNCA, 4 core targets (Figure 6) were obtained. More details of these core targets are illustrated in Table 2.

### 3.8. Results of MD

**Figure 7:**
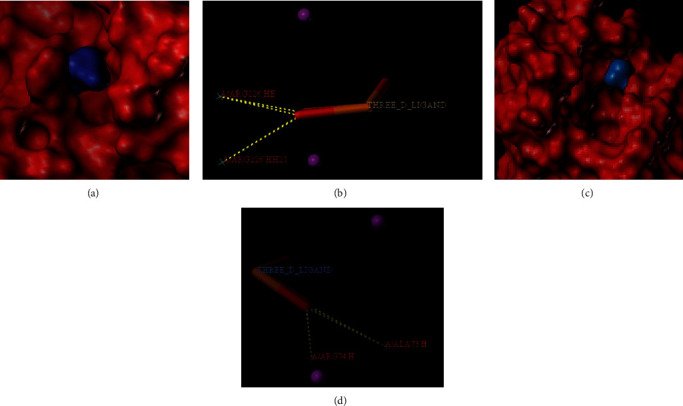
Calcium phosphate: (a, b) CALR protein; (c, d) CALM1 protein.

In our study, CALR, SPARC, CALM1, and CALM2 presented a higher degree. Compared with KEGG enrichment analysis results and UniProt database, we chose 2 target genes, CALR and CALM1, to perform MD experiments. The red part represents the receptor protein. The blue part denotes calcium phosphate. The result indicated that the ingredient, calcium phosphate, enters and binds to active pockets in CALM1 and CALR proteins (Figures 7(a) and 7(c)). The total score was 6.9181 and 5.3811, respectively (Table 3). The yellow dotted line represents the connecting hydrogen bond. Calcium phosphate formed 3 hydrogen bonds with A/ARG226.HE and A/ARG226.HH21 of CALR protein (Figure 7(b)). Simultaneously, calcium phosphate also formed 3 hydrogen bonds with A/ALA73.H and A/ARG74.H of CALM1 protein (Figure 7(d)). These made the ligand (calcium phosphate) and receptors (CALR protein and CALM1 protein) become a stable complex.

## 4. Discussion

Although several western medicines, such as bisphosphonates, calcitonin, and estrogen, generate a good effect on OP, the important role of TCM could not be ignored. It is well-known that TCM has been utilizing in China for over 2500 years [11]. Jintiange capsule, the biomimetic medicine prepared from the artificial tiger bone powders, has been recommended to be the efficient therapy by the treatment guidelines for primary OP and OP-related fracture in China in 2017. We acquired 4 main ingredients of tiger bone including calcium phosphate, magnesium phosphate, osseocolla, and fat from the TCMID database. Among the obtained 4 ingredients, only calcium phosphate has 33 targets retrieved from DrugBank database, and the other ingredients have not discovered the relevant targets. Previous study demonstrated that low phosphate or calcium level or uptake could stimulate vitamin D (cholecalciferol) to undergo position 1 secondary hydroxylation within the kidney. As a result, 1,25-dihydroxyvitamin D (calcitriol), the active metabolite, was formed [12]. It was found in the systemic VDR knockdown model that, the phosphate and calcium-rich diet supplemented with lactose prevented osteomalacia and rickets, thus enhancing calcium uptake by the intestine [13]. The above results suggest the necessity of best extracellular phosphate and calcium contents in order to achieve bone and cartilage mineralization [14].

Then, we predicted disease targets from GeneCards, DisGeNET, and OMIM databases, finally obtaining 4745 disease targets. The drug targets in tiger bone were intersected with disease targets, and 12 overlapping targets were obtained, including CASR, CALR, SPARC, FBN2, RGN, S100A6, TPT1, NUCB2, CALM1, FBN3, CALM2, and NRXN1. Then, we constructed a PPI network of overlapping targets and screened 12 nodes and 25 edges via STRING database, revealing that there existed a complex relationship between these targets.

Subsequently, GO and KEGG pathway analysis were conducted on the overlapping targets. As a result, these overlapping targets were enriched in BP, CC, and MF. According to the results, the jintiange capsule treats OP through many BP, mainly including calcium ion homeostasis, divalent inorganic cation homeostasis, response to metal ion, and others. CC mainly included collagen-containing extracellular matrix, nuclear envelope, spindle, and calcium channel complex. Simultaneously, MF mainly contained calcium channel regulator activity, ion channel binding, cytokine activity, and phosphatase activator activity.

In addition, we observed that 45 pathways were related to these overlapping targets. As revealed by the KEGG enrichment analysis, those identified key targets were mostly associated with Rap1, Ras, cAMP, cGMP-PKG, calcium signaling pathways, and so on. In recent studies, the cGMP-PKG pathway is suggested to modulate the growth and differentiation of osteoblasts by forming the mechanosome that contains Src [15]. As a result, it may exert a critical role in skeletal homeostasis [16]. Rap1 contributes to activating the processes such as clustering of integrin, adhesion of cells onto the bone matrix, transembrane transduction, resultant signal transduction, and cytoskeletal modifications. Rap1 deficiency influences bone resorption in vivo, leading to OP occurrence [17]. The Rap1 pathway determines the osteogenesis or adipogenesis of BMSCs, which clarifies the bone metabolic alterations during OP progression [18]. The increased cAMP/cGMP expression within osteoblasts can induce osteogenesis [19]. RANKL and Cbfa1 contribute to the key factors that maintain bone homeostasis and regulate the differentiation of osteoclasts and osteoblasts, which can be achieved through the cAMP pathway. Angiotensin II and factors such as high/low density lipoprotein (HDL/LDL), homocysteine, and nitric oxide (NO) that are frequently changed in the case of OP and hypertension decreases the Cbfa1 level and increases the RANKL level through the cAMP pathway [20]. Ras pathway has a key function in maintaining the bone stability. After osteocytes are stimulated mechanically, factors related to this pathway are upregulated [21]. Moreover, the Ras pathway modulates immature bone progenitor cell growth and differentiation to osteoblasts, and the activated pathway contributes to activating the downstream PI3K-AKT and MAPK pathways for the enhancement of bone density [22]. The elevation of intracellular calcium levels could activate the calmodulin (CaM)/calmodulin-dependent protein kinase (CaMK) pathway and regulate osteoclast differentiation [23]. According to metabolic analysis and RNA sequencing, the activated Mincle promotes the genesis of osteoclasts by the ITAM-based calcium pathway, which skews the metabolism of osteoclasts to oxidative phosphorylation [24]. Arctiin may suppress the genesis of osteoclasts through inactivating the RANKL-triggered calcium pathway and upregulating the ROS-scavenging enzyme expression related to the Nrf2/Keap1/ARE pathway, which can thus prevent bone loss caused by ovariectomy [25].

As an important research method, D-D-I-T-P networks could better demonstrate the relationship between disease, drug, ingredients, targets, and pathways. In our study, we constructed the current networks. According to the analysis with CytoNCA, we acquired 4 core targets including CALR, SPARC, CALM1, and CALM2.

As the calcium-binding chaperone, CALR targets the calreticulin/calnexin cycle to enhance endoplasmic reticulum (ER) folding, quality control, and oligomeric assembly. Such lectin exhibits transient interaction with nearly every monoglucosylated glycoprotein produced within ER [26]. Additionally, it binds to the NR3C1 DNA-binding domain in order to regulate the transcriptional activity of steroid hormone receptors [27]. CALM1 is responsible for the mediation of L-type calcium channel depending on voltage [28], regulation of CACNA1C inactivation depending on calcium [29], positive regulation of KCNN2's potassium channel activity activated by calcium [30], formation of the KCNQ1-based potassium channel complex, and regulation of channel electrophysiological activities by binding to calcium [31]. Besides, it dominates a role of a sensor for modulating the contact between ER and additional organelles in a VMP1:ATP2A2-mediated manner [32]. Moreover, calmodulin binds to calcium to control numerous ion channels, enzymes, proteins, and aquaporins. Numerous phosphatases and protein kinases are triggered by the calmodulin-calcium complex. Centrin and CCP110 are associated with the genetic pathway regulating centrosome progression and cycle by means of cytokinesis [33]. By performing MD analysis, the main ingredient, calcium phosphate, had good binding with the CALR protein and CALM1 protein. The abovementioned results suggested that the NP-selected major ingredients are reliable and are related to the targets for OP.

## 5. Conclusion

To conclude, this study found that 4 main ingredients of the jintiange capsule, particularly calcium phosphate, could act on 33 targets. Totally, 4745 potential disease targets for OP and 12 overlapping targets were identified by the approach of NP. Then, we constructed a PPI network of these targets, conducted the enrichment analysis of GO and KEGG, constructed D-D-I-T-P networks, obtained 4 core targets, and performed MD experiments to investigate the potential mechanism of the jintiange capsule for treating OP. In summary, the main ingredient of jintiange capsule, particularly calcium phosphate, may interact with 2 targets, CALR and CALM1, and regulate multiple signaling pathways to treat OP. Although these findings based on NP have great significance for understanding the potential mechanism of the jintiange capsule for treating OP and discovering new effective ingredients, there remain some limitations in our study. The obtained findings are only theoretical predictions. In the future, a large number of experiments in vivo or in vitro are still needed to verify these findings.

## Figures and Tables

**Table 1:** Targets of tiger bone's main ingredients.

Main ingredients	Uniprot ID	Protein	Gene symbol
Calcium phosphate	P41180	Extracellular calcium-sensing receptor	CASR
	P27797	Calreticulin	CALR
	Q99828	Calcium and integrin-binding protein 1	CIB1
	O75340	Programmed cell death protein 6	PDCD6
	P30626	Sorcin	SRI
	Q99653	Calcineurin B homologous protein 1	CHP1
	P09486	SPARC	SPARC
	P27824	Calnexin	CANX
	P35556	Fibrillin-2	FBN2
	P04271	Protein S100-B	S100B
	O14958	Calsequestrin-2	CASQ2
	Q15493	Regucalcin	RGN
	Q9UBV8	Peflin	PEF1
	P06703	Protein S100-A6	S100A6
	P13693	Translationally controlled tumor protein	TPT1
	O75838	Calcium and integrin-binding family member 2	CIB2
	Q99584	Protein S100-A13	S100A13
	P31415	Calsequestrin-1	CASQ1
	Q02818	Nucleobindin-1	NUCB1
	P80303	Nucleobindin-2	NUCB2
	P0DP23	Calmodulin	CALM1
	Q75N90	Fibrillin-3	FBN3
	P28676	Grancalcin	GCA
	Q9ULU8	Calcium-dependent secretion activator 1	CADPS
	P0DP24	Calmodulin-2	CALM2
	P0DP25	Calmodulin-3	CALM3
	Q96FQ6	Protein S100-A16	S100A16
	P22676	Calretinin	CALB2
	Q13938	Calcyphosin	CAPS
	Q86UW7	Calcium-dependent secretion activator 2	CADPS2
	Q96L12	Calreticulin-3	CALR3
	Q9ULB1	Neurexin-1	NRXN1
	P62166	Neuronal calcium sensor 1	NCS1

Magnesium phosphate	No ID	No protein	No target
Osseocolla	No ID	No protein	No target
Fat	No ID	No protein	No target

**Table 2:** Information of 4 core targets.

Uniprot ID	Target genes	Protein	Degree
P27797	CALR	Calreticulin	9
P09486	SPARC	SPARC	7
P0DP23	CALM1	Calmodulin-1	7
P0DP24	CALM2	Calmodulin-2	6

**Table 3:** Results of molecular docking of calcium phosphate with 2 proteins.

Ingredient	CALR			CALM1		
	Total score	Crash	Polar	Total score	Crash	Polar
Calcium phosphate	6.9181	−0.3732	7.6852	5.3811	−0.3267	6.9826

## Data Availability

The figures and tables used to support the findings of this study are included within the article, and the original data are available from the first author or corresponding author upon request.
